# Effectiveness of the psychological and pharmacological treatment of catastrophization in patients with fibromyalgia: a randomized controlled trial

**DOI:** 10.1186/1745-6215-10-24

**Published:** 2009-04-23

**Authors:** Javier García-Campayo, Antoni Serrano-Blanco, Baltasar Rodero, Rosa Magallón, Marta Alda, Eva Andrés, Juan V Luciano, Yolanda López del Hoyo

**Affiliations:** 1 Servicio de Psiquiatría, Hospital Miguel Servet y Universidad de Zaragoza, Spain; 2 Sant Joan de Déu-Servicios de Salud Mental; Fundación Sant Joan de Déu. Sant Boi de Llobregat, Barcelona, Spain; 3 Centro Rodero. Clínica de Neurociencias, Santander, Spain; 4 Centro de Salud Arrabal, Zaragoza, Spain; 5 Servicio de Psiquiatría. Hospital de Alcañiz, Teruel, Spain; 6 Statistician University of Zaragoza, Zaragoza, Spain; 7 Grupo Aragonés de Investigación en Atención Primaria, Red de Actividades Preventivas y de Promoción de la Salud (REDIAPP) (G06/170), Instituto Aragonés de Ciencias de la Salud (IACS), Aragon, Spain

## Abstract

**Background:**

Fibromyalgia is a prevalent and disabling disorder characterized by widespread pain and other symptoms such as insomnia, fatigue or depression. Catastrophization is considered a key clinical symptom in fibromyalgia; however, there are no studies on the pharmacological or psychological treatment of catastrophizing. The general aim of this study is to assess the effectiveness of cognitive-behaviour therapy and recommended pharmacological treatment for fibromyalgia (pregabalin, with duloxetine added where there is a comorbid depression), compared with usual treatment at primary care level.

**Method/design:**

*Design*: A multi-centre, randomized controlled trial involving three groups: the control group, consisting of usual treatment at primary care level, and two intervention groups, one consisting of cognitive-behaviour therapy, and the other consisting of the recommended pharmacological treatment for fibromyalgia.

*Setting*: 29 primary care health centres in the city of Zaragoza, Spain.

*Sample*: 180 patients, aged 18–65 years, able to understand and read Spanish, who fulfil criteria for primary fibromyalgia, with no previous psychological treatment, and no pharmacological treatment or their acceptance to discontinue it two weeks before the onset of the study.

*Intervention*: Psychological treatment is based on the manualized protocol developed by Prof. Escobar et al, from the University of New Jersey, for the treatment of somatoform disorders, which has been adapted by our group for the treatment of fibromyalgia. It includes 10 weekly sessions of cognitive-behaviour therapy. Pharmacological therapy consists of the recommended pharmacological treatment for fibromyalgia: pregabalin (300–600 mg/day), with duloxetine (60–120 mg/day) added where there is a comorbid depression).

*Measurements*: The following socio-demographic data will be collected: sex, age, marital status, education, occupation and social class. The diagnosis of psychiatric disorders will be made with the Structured Polyvalent Psychiatric Interview. Other instruments to be administered are the Pain Catastrophizing Scale, the Hamilton tests for Anxiety and for Depression, the Fibromyalgia Impact Questionnaire (FIQ), the EuroQuol-5 domains (EQ-5D), and the use of health and social services (CSRI). Assessments will be carried out at baseline, 1, 3, and 6 months.

*Main variable*: Pain catastrophizing.

*Analysis*: The analysis will be per intent to treat. We will use the general linear models of the SPSS version 15 statistical package, to analyse the effect of the treatment on the result variable (pain catastrophizing).

**Discussion:**

It is necessary to assess the effectiveness of pharmacological and psychological treatments for pain catastrophizing in fibromyalgia. This randomized clinical trial will determine whether both treatments are effective for this important prognostic variable in patients with fibromyalgia.

**Trial registration:**

Current Controlled Trials ISRCTN10804772

## Background

The role of catastrophizing in mediating responses to pain has received considerable attention in recent years [1-3] and a consistent relation between catastrophizing and distress reactions to painful stimulation has been demonstrated [3]. Although the defining criteria for catastrophizing have never been explicitly stated, there is general consensus that this construct involves an exaggerated negative orientation toward noxious stimuli. The aetiology of pain catastrophizing is not clear. It has been demonstrated that interpersonal mechanisms may not play a significant role in its development [4], while insecure attachment would be positively associated with it [5]. Some of the consequences that have been associated with pain catastrophizing are more intense pain [6], heightened pain behaviour [7-9], greater analgesic consumption [10,11], reduced involvement in daily activities [3], occupational disability [12-14] and suicidal ideation [15].

A positive association has been documented between depression and catastrophism [16], but this construct is different from the negative thoughts found in depression. Depressive thoughts are only present associated with depressive mood; however, catastrophism is considered a continuous psychological variable, normally distributed even in healthy individuals without pain or depression [17]. The kinds of cognitions are also different: depressive thoughts are related to depression and similar concepts such as inferiority, guilt or suicide. Catastrophism cognitions are exclusively focused on pain: a negative vision on it (magnification), continuously thinking on it (rumination) and impossibility to control it (helplessness).

A scale to measure catastrophizing, the Pain Catastrophizing Scale (PCS), has been developed and validated [6]. The PCS is a 13-item self-report questionnaire derived partially from the Coping Strategies Questionnaire [16] and other descriptions of catastrophization [1,18,19]. It comprises three dimensions: (a) rumination, (b) magnification and (c) helplessness. Its validity and reliability have been previously reported [6]. Our group has validated the Spanish version of this questionnaire [20].

Fibromyalgia is a prevalent and disabling disorder characterized by a history of widespread pain for at least three months and patient reporting of tenderness in at least 11 of 18 defined tender points when digitally palpated with about 4 kg per unit area of force [21]. Catastrophization is considered a key clinical symptom in fibromyalgia and, in fact, the most used classification of fibromyalgia clinical subtypes includes catastrophization as one of the discriminating variables [22]. However, despite its importance, there is only one study on the psychological treatment of catastrophizing in which the only outcome assessed is the general satisfaction of the patient and his/her knowledge about catastrophizing [23]. Our group has long experience in the treatment of somatoform disorders/fibromyalgia [24,25] and has developed a pioneer psychological treatment of catastrophizing that has proven effective in pilot studies [26].

## Methods/Design

### Objectives

The general aim of the present study is to assess the effectiveness of cognitive-behaviour therapy and recommended pharmacological treatment for fibromyalgia (pregabalin, with duloxetine added where there is comorbid depression), compared with usual treatment at primary care level. The specific objective is to determine the factors (mainly depression and pain) that predict the response of catastrophizing to these treatments.

### Design

**Figure 1:**
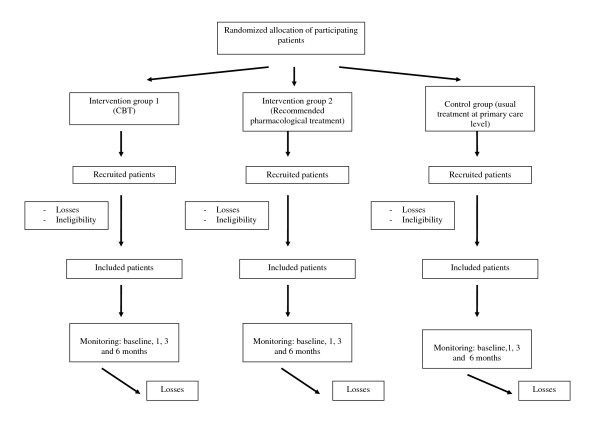
**Flowchart: randomization, sampling and monitoring of patients**.

This is a multi-centre, controlled trial with a random allocation of patients into three alternative branches (see Figure 1):

1. Cognitive-behaviour treatment

2. Recommended pharmacological treatment (pregabalin), associated to antidepressant (duloxetine) if there is a comorbid depression.

3. Treatment as usual at primary care level.

The evaluation of the treatment outcomes will be performed at patient level and they will be assessed individually.

### Setting and study sample

Patients will be recruited from any of the 29 primary health care centres in the city of Zaragoza, Spain. Patients will be recruited by doctors working in these primary care centres until the required sample is completed, without a quota of patients assigned for each centre.

Patients considered for **inclusion **are those aged 18–65 years, able to understand and read Spanish, who fulfil criteria for primary fibromyalgia according to the American College of Rheumatology [21], no previous psychological treatment, no pharmacological treatment or their acceptance to discontinue it two weeks before the onset of the study, and sign informed consent. Those **excluded **will be patients with severe Axis I psychiatric disorders (dementia, schizophrenia, paranoid disorder, abuse of alcohol and/or drug disorders), severe Axis II disorders from the clinician point of view that prevent from following the treatment protocol, pregnancy or lactation, and refusal to participate.

### Randomization, allocation and masking of study groups

Each patient will be allocated to either one of the three groups using a computer-generated random number sequence. The allocation will be carried out by an independent person, belonging to REDIAPP (Research Network on Preventative Activities and Health Promotion), who is not involved in the study. The method used to implement the random allocation sequence will be a central telephone. The sequence will be concealed until interventions are assigned. Patients agree to participate before the random allocation and without knowing which treatment they will be allocated to. Pharmacological treatment will be administered by one psychiatrist (JGC). Study personnel conducting psychological assessments (YLH) will be masked to participants' treatment conditions.

#### Intervention

##### Psychological intervention

Manualized protocol developed by Prof. Escobar et al, from the University of New Jersey, for the treatment of somatoform disorders, which has been adapted by our group for the treatment of fibromyalgia [26]. It includes 10 weekly sessions of cognitive-behaviour therapy.

##### Pharmacological intervention

In this group of patients, pregabalin (300–600 mg/day), the recommended treatment for fibromyalgia will be administered, associated with duloxetine (60–120 mg/day) for the patients in whom a major depression disorder is diagnosed [27]. Pharmacological treatment will be administered and followed-up by a psychiatrist (JGC).

##### Treatment as usual at primary care level

This group will continue usual care at primary care level.

### Measurements

The study personnel carrying out the measurements (RM, MA) will be unaware of which treatment the patients are being administered ("blind"). The follow-up assessments will take place at baseline, 1, 3 and 6 months.

Variables and instruments of measurement (See Table 1)

**Table 1:** Study variables

**Instrument**	**Assessment area Time(s) of assessments**	**Applied by**
Sampling form assistant	Age, sex, inclusion/exclusion criteria Baseline	Research
Sociodemographic data form assistant	Age, sex, marital status, educational level, Baseline	Research
	Socio-economic group [29], occupation	
SPPI psychiatric interview psychiatrist [30]	Psychiatric diagnosis Baseline	Research
Pain catastrophizing scale [20] assistant	Severity of catastrophizing Baseline and follow-up sessions*	Research
Hamilton test for Depression assistant [31,32]	Severity of depression Baseline and follow-up sessions*	Research
Hamilton test for Anxiety assistant [33,34]	Severity of anxiety Baseline and follow-up sessions*	Research
Fibromyalgia Impact assistant Questionnaire [35,36]	General function Baseline and follow-up sessions*	Research
Medical record assistant	Pharmacological Follow-up sessions* side-effect events	Research
EQ-5D [37] assistant	Health related quality of life Baseline and follow-up sessions*	Research
CSRI [38] assistant	Health and social services use Baseline and follow-up sessions*	Research

*Follow-up sessions: 1, 3, and 6 months

#### Main outcome variables

In accordance with the aims of the study, the major outcome is pain catastrophizing in patients with fibromyalgia. This construct will be assessed with the Spanish version [20] of the Pain Catastrophizing Scale [6].

#### Secondary variables

-The following socio-demographic data will be collected: sex, age, marital status (single, married/relationship, separated/divorced, and widowed), education (no studies, primary, lower secondary, upper secondary, university), occupation and social class (I, II, IIIN, IIIM, IV and V of the British Registrar General's Scale) [29].

- The diagnosis of psychiatric disorders will be made with the Structured Polyvalent Psychiatric Interview [30], a psychiatric interview developed by our group and extensively used for the study of somatoform disorders, the group in which fibromyalgia is included in psychiatric classifications.

- Hamilton Anxiety Rating Scale (HARS) [31]. This is a clinician-administered rating scale that consists of 14 items. Each item is rated on a 5-point scale (from 0 = no symptoms to 4 = severe, grossly disabling symptoms. Total scores for the HAS range from 0 to 56. A score of >= 14 has been suggested to indicate clinically significant anxiety. It has a Spanish validated version [32]

- Hamilton Rating Scale for Depression (HAM-D) [33]. This is probably the most used observer-rated depressive symptom rating scale. Although the original scale had 21 items, Hamilton suggested scoring only the initial 17 items because the last 4 items either occurred infrequently or described only aspects of the illness. Items are ranked on a scale of 0–4 (items with quantifiable severity) or 0–2 (items that measure symptoms more difficult to assess reliably). The greatest severity is indicated by 2 or 4. The range for the 17-item scale is 0–50. We have used the Spanish validated version [34].

- Fibromyalgia Impact Questionnaire (FIQ) [35]: The FIQ is a 10-item self-report questionnaire developed to measure the health status of FM patients. The first item focuses on the patient's ability to carry out muscular activities. In the next two items, patients are asked to circle the number of days in the past week they felt good and how often they missed work. Finally, the last seven questions (job ability, pain, fatigue, morning tiredness, stiffness, anxiety and depression) are measured by visual analogue scale (VAS). This instrument has a translated and validated Spanish version [36].

- EuroQoL-5D questionnaire (EQ-5D – Spanish version) [37]: Generic instrument of health-related quality of life. It has two parts: Part 1 records self-reported problems in each of five domains: mobility, self-care, usual activities, pain/discomfort and anxiety/depression. Each domain is divided into three levels of severity corresponding to no problems, some problems, and extreme problems. Part 2 records the subject's self-assessed health on a VAS – a 10-cm vertical line on which the best and worst imaginable health states score 100 and 0, respectively.

- Client Service Receipt Inventory – adapted (CSRI – Spanish version) [38]: Questionnaire for collecting information about use of healthcare and social care services, other economic impacts (such as time off work due to illness) and socio-demographic information. The variant used in this study was designed to collect retrospective data on service utilization during the previous six months.

### Statistical methods

#### Sample size

To calculate the sample size, it is necessary to know the effectiveness of pharmacological and psychological on the main outcome variable, pain catastrophizing. Unfortunately, there are no prior published studies on this subject. Based on our clinical experience, we assume this rate will be by 20% [26]. We aim to detect a difference of 20% or more between any of the groups (control and intervention). Accepting an alpha risk of 0.05 and a beta risk of < 0.20 in a bilateral contrast, we would need 55 patients in each group [28]. Calculating 10% of refusals as found in previous studies [27], we will need a sample size of 60, which implies a total sample of 180 patients with fibromyalgia.

#### Analysis strategy

The analysis will be per intent to treat. First we will compare the three groups in order to verify that there are no significant differences among them (socio-demographic characteristics, clinical baseline data, etc). We will use the mean (standard deviation) in the continuous variables and percentages in the categorical variables. For comparisons we will use the Kruskal-Wallis for continuous variables and the Chi-squared test for categorical variables. Non-parametric tests may also be used.

The main variable of the result is pain catastrophizing. Process variables include socio-demographic characteristics, severity of the depression (Hamilton test for Depression (HAM -D), and anxiety (Hamilton test for Anxiety), and general function (Fibromyalgia Impact Questionnaire).

The general linear models of the SPSS 15 statistical package will be used to analyse the effect of the treatment on the categorical result variables (pain catastrophizing). We will use the analyses of linear mixed models to study the effect of the continuous process variables (depression, anxiety, and pain).

##### Cost-utility analyses

Societal cost perspective will be used for the calculation of costs. Direct costs will be calculated by adding the costs derived from any medication and use of health-related services (general practitioner sessions, specialized medical sessions, emergency room sessions, and hospital in-patient stay). The cost of medications will be calculated by determining the price per milligram during the study, according to the International Vademecum (Red Book) 2008–2010, including value-added tax. Total costs of pharmacological treatment will be calculated by multiplying the price per milligram by the daily dose in milligrams and the number of days such treatment is received. Costs derived from the use of health-related services will be calculated considering the Oblikue unit costs database [39]. Indirect costs will be calculated considering the days of sick leave taken and multiplying them by the minimum daily wage in Spain for 2007–2009. Finally, total costs will be calculated by adding direct and indirect costs.

In Spain, the National Health Service (SNS) is financed by general taxes levied by the state. Medical visits and hospital admissions are fully covered by the SNS. Medications prescribed are fully covered for retired persons, with co-payment for those still in the workforce. Sick leave requires a physician's authorization, and patients unable to work continue to receive most of their salary.

When performing cost-utility analyses, two or more therapeutic options are compared in order to determine which one is the best for maximizing the benefits in light of the available resources [40]. This is achieved by calculating the relationship between the costs of a given intervention (e.g. A) and its consequences, expressed in QALYs, compared with another (e.g. B). This relative value is called incremental cost-utility ratio (ICUR), and it expresses the relationship between the costs and effects of one intervention compared with another.

As the duration of the study is only 12 months, neither costs nor outcomes are subject to discounting [40]. Treatment costs during 12 months follow-up will be modelled by a multivariate gamma regression with a log link. Gamma modelling has been suggested as a suitable choice for analysing cost data, taking into account the skewing of the distribution of the cost data [41,42]. QALYs gained in the first and last six months after the start of the programme will be approximated by measuring the area under the curve using the following equation:



The incremental treatment effect on change in EQ-5D utility score for the first 6 months is d_0–6 _and the incremental treatment effect on change in EQ-5D utility score for the last 6 months is d6-12. These incremental treatment effects will be estimated using multivariate ordinary least squares regressions, adjusting for baseline differences among treatment groups. The covariates included in the models will be: age, sex, years of education, employment and marital status, baseline FIQ and EQ-5D utility scores, and type of medication prescribed. To address uncertainty in the ICUR sampling distribution, non-parametric bootstrapping with five thousand replications will be carried out for each treatment comparison [43].

### Ethical aspects

Informed consent will be obtained from the participants before they are aware of which group they are to be included in. Before they give their consent, the patients will be provided with a general overview of the aims and characteristics of the study and the psychological and pharmacological intervention. They will also be informed that they will be participating voluntarily, and that they can choose to withdraw at any time with the guarantee that they will continue to receive the treatment considered most appropriate by their doctor.

The study follows Helsinki Convention norms and posterior modifications and the Declaration of Madrid of the World Psychiatric Association. The Study Protocol was approved by the Ethical Review Board of the regional health authority (ref: PI07/22).

### Forecast execution dates

Initial recruitment of patients: April 2009

Finalization of patient recruitment: September 2009

Finalization of patient monitoring period: April 2010

Publication of results: September 2010

## Discussion

The effectiveness of pharmacological and psychological treatments for fibromyalgia has been demonstrated, despite the size effect being rather limited [44]. Catastrophizing is considered one of the most important modulating variables in the experience of pain. However, the effectiveness of treatments for fibromyalgia in catastrophizing has not been evaluated except in pilot studies [26].

The strength of the study is that, to our knowledge, this is the first multi-centre, randomized, controlled trial of psychological and pharmacological treatments for pain catastrophizing in patients with fibromyalgia compared with usual treatment.

A number of potential limitations may be difficulties in recruitment, owing to requisite of discontinue pharmacological treatment and changes in employment status because many patients are either on sick leave or applying for disability pensions, making it difficult to interpret the results. The very concept of catastrophizing has also been criticized, with some authors suggesting the need of new tools to assess it [45].

## Competing interests

The authors declare that they have no competing interests.

## Authors' contributions

JGC is the principal researcher and developed the original idea for the study. The study design was further developed by AS, BR and RM. MA, JVL and YLH participated in the design and planning of the intervention that is evaluated here. EA developed the statistical methods. All authors have read and corrected draft versions, and approved the final version.
